# Early Colorectal Cancers Provide New Evidence for a Lynch Syndrome-to-CMMRD Phenotypic Continuum

**DOI:** 10.3390/cancers11081081

**Published:** 2019-07-30

**Authors:** Ceres Fernández-Rozadilla, Miriam Alvarez-Barona, Esther Schamschula, Sahra Bodo, Anael Lopez-Novo, Andres Dacal, Consuelo Calviño-Costas, Angel Lancho, Jorge Amigo, Xabier Bello, Jose Manuel Cameselle-Teijeiro, Angel Carracedo, Chrystelle Colas, Martine Muleris, Katharina Wimmer, Clara Ruiz-Ponte

**Affiliations:** 1 Fundación Pública Galega de Medicina Xenómica SERGAS, Grupo de Medicina Xenómica_USC, IDIS, 15706 Santiago de Compostela, Spain; 2 Division of Human Genetics, Medical University Innsbruck, 6020 Innsbruck, Austria; 3 Department of Biosciences, Paris-Lodron University of Salzburg, 5020 Salzburg, Austria; 4 Sorbonne Université, Inserm, Centre de Recherche Saint-Antoine, CRSA, 75571 Paris, France; 5 Servicio de Gastroenterología, Hospital Universitario Lucus Augusti, IDIS, 27003 Lugo, Spain; 6 Servicio de Pediatría, Hospital Universitario Lucus Augusti, 27003 Lugo, Spain; 7 Servicio de Anatomía Patológica, Hospital Clínico Universitario, USC, 15706 Santiago de Compostela, Spain; 8 Grupo de Medicina Xenómica, Centro de Investigación Biomédica en Red de Enfermedades Raras (CIBERER), Universidade de Santiago de Compostela, 15782 Santiago de Compostela, Spain; 9 Department of Genetics Institut Curie, Centre de Recherche Saint-Antoine, Sorbonne Université, 75571 Paris, France

**Keywords:** Lynch syndrome, CMMRD, phenotypic continuum, genetic modifiers, whole-exome sequencing

## Abstract

Lynch syndrome (LS) is the most common hereditary colorectal cancer (CRC) syndrome, caused by heterozygous mutations in the mismatch repair (MMR) genes. Biallelic mutations in these genes lead however, to constitutive mismatch repair deficiency (CMMRD). In this study, we follow the diagnostic journey of a 12-year old patient with CRC, with a clinical phenotype overlapping CMMRD. We perform molecular and functional assays to discard a CMMRD diagnosis then identify by exome sequencing and validation in a cohort of 134 LS patients, a candidate variant in the *MLH1* UTR region in homozygosis. We propose that this variant, together with other candidates, could be responsible for age-of-onset modulation. Our data support the idea that low-risk modifier alleles may influence early development of cancer in LS leading to a LS-to-CMMRD phenotypic continuum. Therefore, it is essential that larger efforts are directed to the identification and study of these genetic modifiers, in order to provide optimal cancer prevention strategies to these patients.

## 1. Introduction

Lynch syndrome (LS; OMIM 120435) is the most frequent hereditary colorectal cancer (CRC) syndrome that accounts for around 2.8% of cases. It is caused by germline mutations in the mismatch repair (MMR) genes or 3′ deletions in *EPCAM*. LS patients have a 60–80% lifetime risk of developing CRC and other tumours that typically develop around the fifth decade of life and are characterized by microsatellite instability (MSI) and the loss of expression of the corresponding MMR protein [1].

Biallelic MMR germline mutations lead however to constitutive mismatch repair deficiency syndrome (CMMRD; OMIM 276300), characterized by haematologic, brain and colorectal tumours in childhood/adolescence. Diagnostic criteria for the clinical suspicion of CMMRD were defined by the European Care for CMMRD Consortium (C4CMMRD) [2].

However, there is still a current gap in the molecular understanding of MMR-associated phenotypes, and this is reflected in the difficulties in genetic and clinical diagnosis. In this work, we present a male patient from a LS family who developed CRC at 12 years of age. Although he carried a potentially spliceogenic *MSH2* variant in addition to the familial mutation, functional analyses clearly reject CMMRD. Therefore, we evaluated other possible scenarios that could explain the exceptionally early age of CRC onset. Our analyses support the idea of a phenotypic continuum between the classical Lynch and CMMRD syndromes that could be modified by multiple genetic factors.

## 2. Results

*MSH2* predictive testing confirmed that the patient had inherited the pathogenic c.1076+1G>A variant from his mother (Figure S1). Molecular analyses revealed MSI and loss of MSH2/MSH6 expression in the tumour but not in the normal adjacent tissue. A second somatic hit in *MSH2*, c.1035G>A, p. (W345Ter), was found in 42% of tumour reads, together with the original germline variant.

Considering the early presentation, we contemplated the idea that the patient indeed suffered from CMMRD. Germline trio analyses identified only a previously unreported intronic variants of unknown significance (VUS) in *MSH2* (c.1077-11A>G). Although in silico tools predicted a splicing effect, minigene experiments showed that this VUS generates only full-length transcripts (Figure S2), and hence, it is very unlikely the second *MSH2* hit. Furthermore, we could observe neither microsatellite instability (MSI) in DNA from germline (gMSI)/LCL (evMSI), nor the resistance pattern typical of CMMRD-deficient cells (Figures S3 and S4), and hence a molecular CMMRD phenotype was rejected.

Because phenotypes similar to CMMRD can arise from high-penetrance germline mutations in other genes [3], we searched for paternally-inherited or de novo variants in other hereditary CRC genes, but could only find the *MLH1* c.-93G 5′UTR variant in homozygosis (Table S1).

For whole-exome sequencing (WES) analysis, we used the somatic mutational signatures as a proxy to identify pathways relevant to tumorigenesis in this patient. We also looked for tumour mutations in the hereditary cancer genes to check whether the early-onset phenotype could be a result of somatic events, but found no high-impact loss-of-function variants that could account for the phenotype. Interestingly, the tumour mutational pattern was driven by signatures 1, 6, 20, 12, 26 and 3 (Figure S5a). The normal tissue profile was however dominated by signature 12 (Figure S5b). This supports the idea that signature 1 in the cancer (which may seem contradictory with the patient’s young age) is due to the hypermutated nature of the tumour derived from the MSI phenotype. It would also be consistent with the observation of other signatures related to MMR-deficiency (signatures 6, 20 and 26). Additionally, we observed that the tumour somatic mutation burden (TMB, calculated as the total number of mutations/Mb) was 81.8, which is in accordance with a hypermutated tumour expected for LS. The adjacent normal tissue presented a TMB of 18.6.

We then restricted the germline search to genes related to four categories linked to CRC, and found four rare germline candidate variants, including a likely pathogenic non-synonymous change in *IGF1* p. (A118T), and an intronic variant in *FANCC* (c.1073-4G>A) (Tables S2 and S3). We also performed a hypothesis-free rare variant analysis prioritization using eDiVa. Our top hit is a missense variant in the *PTPN4* gene p. (Y126C), yet the *IGF1* p. (A118T) variant still appears at lower rank (Table S4). Because variants in *IGF1* have been reported as LS modifiers, we looked into other previously-described LS modifier genes. Additional candidate modifier variants in *CCND1* p. (P241P) and *MTHFR* p. (R519C) were found (Tables S5 and S6).

A summary of the paternally-inherited candidate variants in this patient can be found on Table 1. These six candidates were selected due to previous evidence supporting their role in cancer risk predisposition, and were genotyped in our LS cohort of 134 LS patients. We only observed variant alleles for *MLH1* and *CCND1*. Albeit non-significant, there was a trend towards a younger age-of-onset in patients with one or more *MLH1* risk alleles (μ_GG_ = 47.66, range (26–85), *n* = 74; μ_GA/AA_ = 44.89, range (25–67, *n* = 46; *p* = 0.112) (Figure S6). *CCND1* analyses did not show evidence for a younger debut.

In parallel, because low-penetrance variants have also been proposed as risk modifiers, we also calculated the polygenic risk score (PRS) to account for the genetic risk explained by low penetrance alleles (Table S7). The patient and his mother exhibited a normalised PRS of 0.408 and 0.472, respectively, which fall into the population-expected range (percentiles 47 and 49), so although some of these variants could be individual risk modifiers, we cannot link the early onset observed in this patient with an outstanding contribution of lower penetrance alleles.

## 3. Discussion

In this work, we present a LS patient who exhibits an extremely early age-of-onset of CRC at only 12 years. We initially hypothesized that the patient could have CMMRD, and identified a likely spliceogenic intronic VUS in *MSH2*. Although we cannot fully exclude other explanations (a low-frequency mosaic mutation in the healthy colonic mucosa or a hypomorphic leaky splice effect of the VUS not revealed by the minigene experiments), the molecular analyses performed do not support a CMMRD molecular phenotype and diagnosis.

We then hypothesised that the presence of other high/moderately penetrant mutations may be responsible for the early onset of cancer [4]. Nevertheless, we could only find a variant in the 5′UTR of the *MLH1* gene in homozygosis, which could have important consequences on the risk of developing CRC (odds ratio 1.3 and 2.6 for heterozygous and homozygous, respectively, Thomas R et al. under review). This variant has been extensively reported in the literature as a low-penetrance allele in MSI cancers [5], as it is related to the epigenetic regulation of the *MLH1* CpG island and shore [6]. We find that there is a non-significant trend towards a younger age of onset in patients carrying the risk variant in our cohort of LS patients. Notably, there was another 24-year-old patient in our LS cohort that presented a CMMRD-overlapping phenotype, who presented the *MLH1* variant in homozygosis.

Subsequently, we considered other genes described as genetic modifiers of LS, and found interesting variants in *IGF1*, *CCND1*, *MTHFR, FANCC* and *PTPN4*. The *IGF1* gene codes for a growth factor determinant in cell cycle control. Variants in this gene have been linked to an early onset of CRC in LS [7].

Cyclin D1 also plays a relevant role in cancer, and the variant found in this patient codes for a synonymous change that affects splicing [8] and has been related to abnormal cell proliferation [9]. This SNP has been extensively studied in the context of LS, although with ambiguous results [10,11]. The *MTHFR* gene codifies for the rate-limiting enzyme that regulates folate availability and two of its most common SNPs are low penetrance alleles for CRC [12,13]. Lastly, *PTPN4* belongs to the superfamily of protein tyrosine-kinases and phosphatases, frequently mutated in cancers. Although not much is known about *PTPN4*, it has been suggested that mutations in another family member, *PTPN12*, could cause susceptibility to CRC [14]. Finally, *FANCC* belongs to the Fanconi anaemia DNA repair pathway, which has been proposed to play a role in inherited predisposition to CRC [15].

Altogether, we propose that the combination of several low-risk modifier alleles may be responsible for the CMMRD phenotypic overlap in this patient. The presence of these or other genetic modifiers could potentially explain the higher prevalence of childhood cancers in LS patients [16]. Nevertheless, we cannot exclude that other epigenetic or environmental factors may also play a role in early CRC development.

## 4. Materials and Methods

We initially studied a patient from a LS family who developed CRC at 12 years of age. Upon colonoscopy and histological analyses, the presence of a stage II adenocarcinoma in the caecum (T2N0M0) was confirmed. Additional clinical findings compatible with a CMMRD diagnosis included a neurofibroma in the back (although not histologically confirmed). No café-au-lait macules (CALMs) were observed. The patient and his family received informed consent, according to the tenets of the Declaration of Helsinki, and then *MSH2* predictive testing and somatic sequencing of the complete coding region of *MSH2* were performed to confirm the LS diagnosis. Multi-gene targeted sequencing and multiplex ligation-dependent probe amplification (MLPA) were used to identify other potential germline (epi)mutations in other cancer susceptibility genes: *MSH6*, *MLH1*, *PMS2*, *APC*, *MUTYH*, *POLE*, *POLD1* and *NF1*. In silico splicing predictor analyses and minigene assays were performed to evaluate the splicing effect of the *MSH2* variant of unknown significance (VUS) c.1077-11 A>G. Germline and ex vivo MSI, and toxicity tolerance to MNNG were additionally assayed to evaluate MMR deficiency (see online information for detailed description of these assays).

Whole-exome sequencing (WES) was performed from blood germline DNA for the patient and both parents, to identify the variants responsible for the early onset. Additionally, the tumour and matched normal colonic tissue were also analysed to characterize somatic mutation features. Median coverage was 55× and 150× for the germline and somatic tissues. An additional resequencing panel with Ion PGM was also performed on the *MSH2* gene. This panel yielded an average sequencing depth in the normal somatic tissue of 3225×, which would be adequate for somatic mosaicism analyses if we consider a variant allele frequency (VAF) of 10% as a threshold for somatic mosaicism calling. We selected either de novo or paternally-inherited alleles, with loss-of-function, high functional impact or known modifier effects as candidates. All candidate changes were confirmed by Sanger sequencing. The EDiVa bioinformatics tool (https://ediva.crg.eu/) was used to obtain a variant list ranked by potential pathogenicity.

We validated the effect of these candidate variants on age of CRC onset by genotyping in a cohort of 134 LS patients (26 with pathogenic variants in *MLH1*, 88 in *MSH2* and 20 in *MSH6*; age range 25–85).

Because it has been described that low-penetrance alleles may also act as genetic modifiers of CRC risk, we also genotyped the 37 GWAS-described non-exonic risk variants that had attained genome-wide significance (*p* value ≤ 5 × 10^−8^) to generate the polygenic risk score (PRS) (see online material). The mutational signature in the tumour was obtained with MuSiCa [17]. Because LS tumours are expected to be hypermutated, we assessed the mutational burden as the number of somatic mutations/megabase. A detailed description of the methods is found in Appendix A.

## 5. Conclusions

We recommend that patients with a clinical CMMRD-overlapping phenotype be subject to molecular testing to discard CMMRD. Then, further efforts should be made, given the current genomics era, into the identification of modifier genes and variants. In this regard, although there have been studies trying to identify LS modifiers for the past 20 years [18,19], these have yielded inconsistent results. We must make an effort in order to design robust studies with appropriate sample sizes that can assess the effects of these genetic modifiers on age of onset. All of these could prove effective in finally bringing the search for modifier alleles in the Lynch-to-CMMRD phenotypic continuum forward.

## Figures and Tables

**Table 1:** Germline candidate variants. Germline changes found by exome analysis that could potentially have a modifying effect in the LS environment and could therefore affect age of cancer onset.

Chromosome: Position	Reference Allele	Alternative Allele	Genotype	Gene	Variant	Location	HGMD	Automated InterVar	dbSNP ID	gnomAD NFE	CADD Phred	DANN Score	GERP++ Score	Interpro Domain
1:11852412	G	A	het	*MTHFR*	NM_005957:c.1555C>T,	exonic	-	Uncertain significance	rs45496998	0	34	0.999	4.19	-
					p. (R519C)									
2:120639370	A	G	het	*PTPN4*	NM_002830:c.A377G,	exonic	-	Uncertain significance	NA	0	6.13	0.998	5.41	-
					p. (Y126C)									
3:37034946	G	A	hom	*MLH1*	NM_000249:c.-93G>A *	UTR5	DFP	-	rs1800734	0.222				-
9:97876996	C	T	het	*FANCC*	NM_000136:c.1073-4G>A	splicing/intronic	-	-	rs147695697	0	-	-	-	-
11:69462910	G	A	het	*CCND1*	NM_053056:c.723G>A, p.(P241P)	exonic	DFP	Benign	rs9344	0.465	-	-	-	-
12:102813337	C	T	het	*IGF1*	NM_000618:c.352G>A,	exonic	-	Likely pathogenic	rs151098426	0.001	24.1	0.998	5.85	Insulin, conserved site
					p. (A118T)									

Hom: homozygote; het: heterozygote; HGMD: Human Gene Mutation Database class; DFP: Disease-associated polymorphism with additional supporting functional evidence; frequency: gnomAD NFE variant frequency in gnomAD all in non-Finish Europeans; CADD; DANN, GERP++: in silico predictors of pathogenicity. All variants are paternally-inherited with the exception of *, where mother and father are heterozygous. No de-novo variants were found that fulfilled our prioritization criteria.

## Supplementary Materials

The following are available online at https://www.mdpi.com/2072-6694/11/8/1081/s1. Table S1: Variants in hereditary CRC genes, Table S2: Candidate genes for the rare variant analysis, Table S3: List of rare variants in candidate genes from the WES germline analyses, Table S4: Candidate variant prioritization with eDiVa, Table S5: Modifier genes in LS, Table S6: List of variants found in the described Lynch syndrome modifier genes, Table S7: GWAS loci used for PRS score, Figure S1: Family pedigree, Figure S2: Minigene experiments, Figure S3: Ex vivo MSI assays, Figure S4: Methylation tolerance assays, Figure S5: Mutational signature of the somatic tissues, Figure S6: Comparison of age of onset relative to the MLH1 c-93 G>A variant genotype.

## Appendix A

### Appendix A.1. Ethics

Informed consent (as described in the tenets of the Declaration of Helsinki) was obtained from all patients.

### Appendix A.2. LS Diagnosis

Germline DNA was obtained from a peripheral blood sample using the Chemagic DNA blood kit (Chemagen Biopolymer-Technologie AG, Baesweiler, Germany). For immunohistochemistry, the tissue specimens were fixed in phosphate-buffered, 10% formalin and included in paraffin blocks. Paraffin-embedded sections were stained with H&E. The immunohistochemical studies were performed on 3-μm-thick paraffin sections using a peroxidase-conjugated labeled dextran polymer (EnVision FLEX/HRP; Dako, Glostrup, Denmark), with 3,3′-diaminobenzidine as the chromogen and using a platform (Autostainer Link 48, Dako). The primary antibodies were used as follows: MSH2 (clone FE11, ready-to-use, high pH; Dako); MSH6 (clone EP49, ready-to-use, high pH; Dako); MLH1 (clone ES05, ready-to-use, high pH; Dako); and PMS2 (clone EP51, ready-to-use, high pH; Dako). Tumour and matching normal bowel DNAs were also extracted from the FFPE embedded tissue with the GeneRead DNA FFPE kit (Qiagen, Hilden, Germany). Predictive Sanger bidirectional sequencing was performed for *MSH2* exon 6 on an ABI ABI3730 instrument (Applied Biosystems, Carlsbad, CA, USA) to check for carrier status of the c.1076+1G>A pathogenic variant in the patient, then the whole exonic sequence was analysed to identify the second *MSH2* somatic event in the tumour.

### Appendix A.3. CMMRD Diagnosis

The genes *MSH2*, *MSH6*, *MLH1*, *PMS2*, *APC*, *MUTYH*, *POLE*, *POLD1* and *NF1* were sequenced either by bidirectional Sanger sequencing and/or in the Ion Proton platform (Thermo Fisher, Waltham, MA, USA) to identify additional germline point mutations. Identified variants were validated by bidirectional Sanger sequencing. Multiplex ligation-dependent probe amplification (MLPA) was used to evaluate the presence of germline exonic duplications/deletions in hereditary CRC syndrome genes (SALSA MLPA P003-*MLH1* & *MSH2*; P072-*MSH6*; P008-*PMS2*; P043-*APC*; P378-*MUTYH*) and MMR inactivation via methylation (SALSA MLPA ME011-Mismatch Repair genes). This latter includes probes to test aberrant CpG island methylation of *MLH1*, *MSH2*, *MSH6*, *PMS2*, *MSH3* and *MLH3*. For all purposes, the *MSH2* reference used is NM_000251.2. Splicing predictor tools such as SpliceSiteFinder-like, NNSPLICE or MaxEnt in Alamut (Interactive Biosoftware, Rouen, France) were used to predict the pathogenicity of the c.1077-11 A>G variant.

### Appendix A.4. Minigene Assays

The RTB hybrid-minigene plasmid [20] was used to construct minigenes to determine the splice effect of the c.1077-11 A>G variant in exon 7 of *MSH2*. Exon 6 and 7 with flanking intronic sequences were amplified from genomic DNA obtained from the patient using primers introducing *Sal*I and *Kpn*I sites at the 5′ and 3′ ends respectively. The resulting amplicons were cloned into the internal exon 2 and the last, exon 4, of the RTB minigene where *Sal*I and *Kpn*I sites are located. Clones containing the plasmid with wildtype and mutant insert were selected by colony PCR and subsequent sequencing. The integrity of all constructs was confirmed by sequencing of plasmid maxi-preparations prior to transient transfection in HEK293, HeLa, HRT-18 and SH-SY5Y cell lines. Cells were transfected with 4 μg of plasmid DNA using Turbofect reagent (Thermo Scientific, Waltham, MA) following the manufacturer’s protocol. RNA was extracted with RNeasy Mini Kit (Qiagen, Limburg, The Netherlands) 48 h after transfection. For cDNA synthesis, 1 μg of RNA was reverse transcribed using random hexamers and High Capacity RNA-to-cDNA Kit (Applied Biosystems, Carlsbad, CA, USA). Transcripts of the hybrid RTB minigenes were PCR-amplified using primers located in exon 1 and 4 of the plasmid. The PCR products were examined on 2.0% TAE-agarose gels containing ethidium bromide and sequenced with primer RTBP4F.

### Appendix A.5. Germline and Ex Vivo MSI

Germline microsatellite instability (gMSI) was evaluated and analysed by the methodology described by Ingham et al. [21]. Briefly, DNA extracted from patient´s PBLs was used to amplify three dinucleotide microsatellite repeats (D17S791, D2S123 and D17S250) to detect the presence of stutter peaks. In parallel, a lymphoblastoid cell line (LCL) was established from the patient. We compared electropherograms of PCR products from LCL and peripheral blood lymphocytes (PBLs) following amplification of the NR27, NR21 and BAT26 mononucleotide microsatellites in order to evaluate ex vivo MSI [22].

### Appendix A.6. Methylation Tolerance Assays

Exponentially growing LCLs from the patient were exposed to 2 or 3 pulses of 1.2, 2.5 or 5 μM *N*-Methyl-*N*-Nitro-*N*-Nitrosoguanidine (MNNG) as described in Bodo et al. 2015 [22]. Briefly, cytotoxicity was examined with the WST kit according to manufacturer´s instructions (Roche, Indianapolis, IN, USA). The percentage of cell survival was represented as the absorbance of treated sample relative to control as measured at 450 nm in a microplate Tecan Infinite F500 reader (Tecan, Männedorf, Switzerland).

### Appendix A.7. Exome Sequencing (WES)

We performed germline WES for the patient and both parents. DNA for the latter was obtained from a peripheral blood sample, as explained in a previous paragraph for the patient sample. Libraries were prepared with the Exome Capture Nimblegen SeqCap v3 (64 Mb) kit (100 bp paired-end reads) and sequenced on an Illumina HiSeq2000 platform to achieve a median coverage of 55×. Reads were then aligned to the GRCh37 reference sequence with the GEM algorithm. Variant calling was performed using SamTools, GATK HaplotypeCaller, Pindel and SNAPE, and annotation was done with SnpEff v3.6 [23,24,25,26,27]. Read QC included trimming of the adaptors, and read trimming based on base quality (particularly in the 3′end). Variant QC was performed by filtering by QUAL ≥30, a minimum depth per variant of 30 and removal of variants with strand bias <0.05. Additionally, to reduce artifacts, we further excluded all variants with <20% variant reads and/or <4 variant reads. Loss of function mutations (nonsense, frameshift and splicing on ±1/2) were selected, together with high functional impact missense variants (CADD_phred >10, DANN >0.9 and GERP++ > 3). For rare variant analysis, a filter of <1% MAF in gnomAD was used. Exome Disease Variant Discovery (eDiVa-http://ediva.crg.eu/) was used to obtain a variant list ranked by potential pathogenicity for paternally-inherited dominant, recessive and de novo models of inheritance. All of the reported variants are actually paternally-inherited changes as no de novo variants were found that fulfilled our prioritization criteria.

### Appendix A.8. Modifier Effect on Age

In order to estimate the effect that these gene modifiers could have on age of onset, we inspected the age distribution with regards to modifier variant phenotypes in a cohort of 134 known LS patients with identified mutations in *MLH1* (19%), *MSH2* (66%) or *MSH6* (15%). All patients had received informed consent and the procedure had been approved by the corresponding ethical board, in accordance with the tenets of the Declaration of Helsinki. Variant population allele frequencies were based on genomeAD and in the Collaborative Spanish Variant Server [28]. Genotypic deviations from Hardy–Weinberg equilibrium were also calculated for each marker.

### Appendix A.9. Polygenic Risk Score (PRS)

Given the fact that some of our findings point to low-penetrance alleles as potential genetic modifiers of age of onset of cancer in LS patients, we decided to explore the genetic landscape of the common risk variants in the patient as compared to his mother. For this purpose, we generated a polygenic risk score (PRS) based on the 37 susceptibility SNPs that have been described by genome-wide association studies (GWAS). Because the risk increase conferred by each of these variants is so small, the PRS aims to provide a quantitative score of susceptibility caused by common genetic variants [29]. The risk variants were genotyped with the Sequenom MassARRAY technology (Agena Bioscience, San Diego, CA, USA). Then, we generated the PRS profile for the trio as:

$$\mathrm{Log}\overset{37}{\underset{i=1}{\sum }}\left(\beta \mathrm{iNi}\right)$$

where β = Ln (reported odds ratio), for each risk allele and N is the number of risk alleles. Hemizygous calls for males in the X chromosome (for rs5934683) were considered as homozygous. The scores were then transformed into a 0–1 scale for normalization and easier interpretation.

### Appendix A.10. Somatic WES

Additionally, WES from the somatic tumour tissue and the matched normal mucosa from the same paraffin block were also performed with the SureSelect Human All Exon V6 kit (Agilent Technologies, Santa Clara, CA, USA) ran on an Ion Proton™ system (Life Technologies, Carlsbad, CA, USA). Tumour heterogeneity was accounted for by adjusting the heterozygous calls to variants that were present in at least 15% of the reads. Then, we selected all regions with ≥30× read depth in both samples. Additionally, we removed every variant present at >80% depth in the tumour to exclude potential germline variants that had been missed. The list of tumour exclusive mutations was produced by removing those present in the normal tissue and excluding low-quality variants and sequencing artefacts with the pipeline described for the Cancer Genome Atlas (TCGA) somatic variant calling using VarScan https://docs.gdc.cancer.gov/Data/Bioinformatics_Pipelines/DNA_Seq_Variant_Calling_Pipeline/#somatic-variant-calling-workflow). Additionally, variants present at >2× in ExAC were also removed, as per the latest recommendations on TMB calculations [30]. Because FFPE samples are prone to sequencing errors due to the low quality of the DNA, we performed a validation via Sanger sequencing of 20 variants in both the normal and tumour tissues. These variants were selected based on the most predominant changes expected to appear in FFPE tissues due to embedding. We observed error rates of 21% for the tumour and 17% for the normal somatic tissues and hence down-sampled the variant files accordingly.

We inspected the presence of mutational signature contributions in the tumour, as described by Alexandrov et al. [31], with the help of the MuSiCa software [17]. Because LS tumours are expected to be hyper-mutated due to the impaired ability to repair DNA, we also assessed the mutational burden in the tumour, as the number of somatic mutations per megabase.
