# Supplementary material for: Early Colorectal Cancers Provide New Evidence for a Lynch Syndrome-to-CMMRD Phenotypic Continuum
Source: Cancers (Basel). 2019 Jul 30;11(8):1081. doi: 10.3390/cancers11081081 (PMC6721314; doi:10.3390/cancers11081081)
Supplement: Supplementary file 1 [file cancers-11-01081-s001.pdf]

Supplementary Material

Early Colorectal Cancers Provide New Evidence for a Lynch Syndrome-to-CMMRD Phenotypic Continuum

Ceres Fernandez-Rozadilla, Miriam Alvarez-Barona, Esther Schamschula, Sahra Bodo, Anael Lopez-Novo, Andres Dacal, Consuelo Calviño-Costas, Angel Lancho, Jorge Amigo, Xabier Bello, Jose Manuel Cameselle-Teijeiro, Angel Carracedo, Chrystelle Colas, Martine Muleris, Katharina Wimmer and Clara Ruiz-Ponte

Supplementary Tables

Table S1. Variants in hereditary CRC genes. List of paternally-inherited variants present in the genes related to CRC hereditary syndromes. *mother and father are heterozygous. No de-novo variants were found that fulfilled our prioritization criteria.

Chromosome	Start	End	Reference Allele	Alternative Allele	Genotype	Gene	Variant	Location
2	47656870	47656870	A	G	het	*MSH2*	NM_000249.3:c.1039-29A>T*	intronic
2	48010654	48010654	C	G	het	*MSH6*	NM_000179.2:c.260+22C>G	intronic
**3**	**37034946**	**37034946**	**G**	**A**	**hom**	***MLH1***	**NM_000249:c.-93G>A***	**UTR5**
5	112113527	112113527	A	T	het	*APC*	NM_000038.4:c.531+2093A>T*	intronic
12	133252796	133252796	C	G	het	*POLE*	NM_006231.3:c.910-6G>C	intronic
12	133253310	133253310	A	G	het	*POLE*	NM_006231.3:c.802-71T>C	intronic
12	133254083	133254083	T	C	het	*POLE*	NM_006231.3:c.721-54A>G	intronic
12	133256904	133256904	C	T	het	*POLE*	NM_006231.3:c.286-96G>A	intronic

Table S1. Continued.

Chromosome	Start	End	ClinVar/HGMD	SNP ID	gnomAD NFE
2	47656870	47656870		rs6771325	0.035
2	48010654	48010654	Benign	rs55927047	0.184
**3**	**37034946**	**37034946**	**Benign/DFP**	**rs1800734**	**0.222**
5	112113527	112113527	other	rs467033	0.484
12	133252796	133252796		rs4077170	0.699
12	133253310	133253310		rs5744757	0.571
12	133254083	133254083		rs5744750	0.571
12	133256904	133256904		rs5744738	0.411

Table S2. Candidate genes for the rare variant analysis. Variants with MAF <1% in the hereditary cancer genes (as included in the TruSight panel-https://www.illumina.com/content/dam/illumina-marketing/documents/products/gene_lists/gene_list_trusight_cancer.xlsx) and our own clinical diagnostic panel, were selected, together with those in the following KEGG categories (ref): (i) base-excision repair (BER-hsa03410); (ii) direct reversal (DR-hsa03400); (iii) Fanconi anaemia (hsa03460); (iv) homologus recombination (HR-hsa03440); (v) mismatch repair (MMR-hsa03430); vi) nucleotide excision repair (NER-hsa0342); (vii) non-homologous end joining (NHEJ-hsa03450); (viii) TGF-beta pathway (hsa04350); (ix) Wnt signaling pathway (hsa04310); (x) Pathways in cancer (hsa05200) [1–4].

Category	Genes
Hereditary cancer genes	*AIP, ALK, APC, ATM, AXIN2, BAP1, BARD1, BLM, BMPR1A, BRCA1, BRCA2, BRIP1, BUB1, BUB3, CDC73, CDH1, CDK4, CDKN1C, CDKN2A, CEP57, CHEK2, CYLD, DDB2, DICER1, DIS3L2, EGFR, ENG, EPCAM, ERCC2, ERCC3, ERCC4, ERCC5, EXO1, EXT1, EXT2, EZH2, FAN1, FANCA, FANCB, FANCC, FANCD2, FANCE, FANCF, FANCG, FANCI, FANCL, FANCM, FH, FLCN, FOCAD, GATA2, GPC3, GREM1, HNF1A, HRAS, KIT, LRP6, MAP3K6, MAX, MEN1, MET, MLH1, MLH3, MSH2, MSH3, MSH6, MUTYH, NBN, NF1, NF2, NSD1, NTHL1, PALB2, PHOX2B, PMS1, PMS2, POLD1, POLE, PRF1, PRKAR1A, PTCH1, PTEN, PTPN12, RAD51C, RAD51D, RB1, RECQL4, RET, RHBDF2, RPS20, RUNX1, SBDS, SDHAF2, SDHB, SDHC, SDHD, SLX4, SMAD4, SMAD9, SMARCB1, STK11, SUFU, TMEM127, TP53, TSC1, TSC2, VHL, WRN, WT1, XPA, XPC, XPV*
Linkage	*5S_rRNA, 7SK, A4GNT, AAED1, AB586698, ABCB10, ABTB1, ACAD11, ACAD9, ACBD3, ACPP, ACTA1, ACTN2, ADCK3, ADCY5, ADIPOR1, ADORA1, AGT, AIDA, AK025140, AK054726, AK055533, AK055856, AK056351, AK057451, AK092251, AK094916, AK095132, AK095633, AK097184, AK123177, AK124056, AK124970, AK125726, AK127087, AK128202, AK304483, AK309476, AK310239, AK316092, AL137535, ALDH1L1, ALDH1L1-AS1, ALDH1L1-AS2, ALG1L, ALG1L2, ALG2, AMOTL2, ANAPC13, ANGEL2, ANKRD19P, ANKS6, ANP32B, ARF1, ARGFX, ARID4B, ARL8A, ARMC8, ARV1, ASCL5, ASPN, ASTE1, ATF3, ATP2B4, ATP2C1, ATP6V1G3, AUH, AURKAPS1, AVPR1B, AX229788, AX746764, AX746834, AX746877, AX746920, AX747026, AX747081, AX747246, AX747261, AX747377, AX747652, AX748016, AX748369, B3GALNT2, BARX1, BATF3, BC007984, BC010168, BC015435, BC015846, BC016143, BC016656, BC016972, BC032040, BC032899, BC032911, BC033346, BC033989, BC034684, BC037929, BC038725, BC038769, BC039356, BC039686, BC040684, BC040869, BC041030, BC042374, BC042913, BC042955, BC045735, BC049825, BC070371, BC080653, BC171896, BFSP2, BICD2, BPESC1, BPNT1, BROX, BTG2, BX537548, BX537742, BX641154, C1orf106, C1orf115, C1orf116, C1orf131, C1orf140, C1orf145, C1orf186, C1orf198, C1orf227, C1orf35, C1orf65, C1orf74, C1orf81, C1orf95, C3orf22, C3orf27, C3orf36, C3orf37, C3orf56, C3orf72, C4BPA, C4BPB, C9orf102, C9orf129, C9orf156, C9orf3, C9orf47, C9orf89, CABC1, CACNA1S, CAMK1G, CAMSAP2, CAPN2, CAPN8, CAPN9, CASR, CCDC14, CCDC180, CCDC37, CCDC58, CCRL1, CCSAP, CD34, CD46, CD55, CD86, CDC14B, CDC42BPA, CDK18, CDK20, CDV3, CENPF, CENPP, CEP63, CEP70, CHCHD6, CHI3L1, CHIT1, CHST13, CKS2, CLDN18, CLSTN2, CNBP, CNIH3, CNIH4, CNTN2, COA6, COG2, COL15A1, COL6A4P2, COL6A5, COL6A6, COPB2, COPG1, CORO2A, CPNE4, CR1, CR1L, CR2, CSRP1, CSTA, CTSE, CTSL1, CTSL1P8, CTSL2, CTSL3P, CYB5R1, DAPK1, DBR1, DDX59, DEGS1, DIEXF, DIRAS2, DIRC2, DISC1, DISC2, DISP1, DKFZp434B1222, DL490795, DM119500, DM119504, DM119532, DNAH14, DNAJB8, DNAJB8-AS1, DNAJC13, DNAPTP1, DQ570601, DQ572301, DQ573170, DQ573684, DQ574659, DQ574660, DQ575010, DQ575955, DQ575983, DQ576410, DQ576681, DQ578031, DQ578098, DQ579267, DQ579694, DQ579969, DQ583119, DQ584609, DQ584857, DQ584939, DQ584971, DQ584993, DQ585001, DQ586768, DQ587539, DQ587746, DQ587965, DQ588075, DQ590432, DQ590442, DQ590589, DQ593488, DQ594798, DQ596206, DQ596352, DQ597036, DQ597235, DQ597892, DQ598665, DQ599768, DQ599799, DQ599872, DQ600234, DQ601751, DQ673941, DQ786258, DSTYK, DTL, DTX3L, DUSP10, DUSP5P1, DYRK3, DZIP1L, EAF2, ECM2, EDARADD, EEFSEC, EFCAB12, EFCC1, EGLN1, EIF2D, ELF3, ELK4, ENAH, EPHB1, EPHX1, EPRS, ERCC6L2, ERO1LB, ERP44, ESRRG, ESYT3, ETNK2, EU250746, EXOC8, FAIM, FAIM3, FAM120A, FAM120AOS, FAM162A, FAM177B, FAM71A, FAM72A, FAM86HP, FAM86JP, FAM89A, FANCC, FBP1, FBP2, FBXO28, FBXO40, FCAMR, FGD3, FLVCR1, FLVCR1-AS1, FMOD, FOXE1, FOXL2, G0S2, GABBR2, GADD45G, GALNT12, GALNT2, GATA2, GGPS1, GJC2, GNG4, GNPAT, GOLGB1, GOLT1A, GP9, GPATCH2, GPR137B, GPR25, GPR37L1, GUK1, H1FOO, H1FX, H1FX-AS1, H3F3A, H3F3AP4, HABP4, HCLS1, HEATR1, HEG1, HEMGN, HHAT, HHIPL2, HIATL1, HIATL2, HIST3H2A, HIST3H2BB, HIST3H3, HLX, HM358976, HSD11B1, HSD17B3, HSPBAP1, Histone3, IARS, IARS2, IBA57, IFT122, IGFN1, IKBKE, IL10, IL19, IL20, IL20RB, IL24, ILDR1, INTS7, INVS, IPO9, IPPK, IQCB1, IRF2BP2, IRF6, ISY1, ISY1-RAB43, ITGB5, ITPKB, JB148981, JB149101, JB153432, JMJD4, KALRN, KBTBD12, KCNH1, KCNK1, KCNK2, KCTD3, KDM5B, KDM5B-AS1, KIAA1257, KIAA1804, KIF14, KIF21B, KISS1, KLF15, KLHDC8A, KLHL12, KPNA1, KY, L25629, LAD1, LAMB3, LAX1, LBR, LEFTY1, LEFTY2, LEMD1, LEMD1-AS1, LGALS8, LGALS8-AS1, LGR6, LIN9, LINC00092, LINC00184, LINC00210, LINC00260, LINC00303, LINC00467, LINC00475, LINC00476, LINC00538, LINC00582, LINC00628, LINC00862, LMOD1, LOC100128076, LOC100128361, LOC100129316, LOC100129550, LOC100130093, LOC100132077, LOC100132781, LOC100499484, LOC100499484-C9ORF174, LOC100506795, LOC100506810, LOC100507032, LOC100507346, LOC148696, LOC148709, LOC149373, LOC158434, LOC158435, LOC284578, LOC284581, LOC286238, LOC286359, LOC286370, LOC339874, LOC340508, LOC340515, LOC392364, LOC401980, LOC441454, LOC441455, LOC441461, LOC641515, LOC643723, LOC653712, LOC728463, LOC730227, LOC90246, LPGAT1, LRRN2, LYPLAL1, LYST, MAP10, MAPKAPK2, MARK1, MBD4, MCM2, MDM4, MFSD4, MGLL, MIA3, MIR1182, MIR1231, MIR135B, MIR1537, MIR181A1, MIR181A1HG, MIR181B1, MIR194-1, MIR205, MIR205HG, MIR215, MIR2278, MIR23B, MIR29C, MIR3074, MIR3122, MIR3153, MIR320B2, MIR3620, MIR3910-1, MIR3910-2, MIR4260, MIR4289, MIR4290, MIR4291, MIR4427, MIR4666A, MIR4670, MIR4671, MIR4742, MIR4753, MIR4788, MIR5002, MIR5008, MIR5092, MIR5191, MIR548I1, MIR5704, MIRLET7A1, MIRLET7D, MIRLET7DHG, MIRLET7F1, MIXL1, MRAS, MRPL3, MRPL55, MRPS22, MSL2, MT1HL1, MTR, MUC13, MYBPH, MYLK, MYLK-AS1, MYOG, Metazoa_SRP, Mir_340, Mir_384, Mir_544, Mir_598, NAMA, NAMA_1, NAMA_2, NANS, NAV1, NCBP1, NCK1, NEK11, NEK2, NENF, NFASC, NFIL3, NID1, NINJ1, NME9, NMNAT3, NOL8,*

	NPHP3, NPHP3-ACAD11, NPHP3-AS1, NR4A3, NR5A2, NSL1, NTPCR, NUAK2, NUCKS1, NUDT16, NUDT16P1, NUP133, NUP210P1, NUTM2F, NUTM2G, NVL, NXNL2, OBSCN, OGN, OMD, OPTC, OSBPL11, PARP1, PARP14, PARP15, PARP9, PCCB, PCNXL2, PDIA5, PFKFB2, PGBD5, PHF2, PHLDA3, PIGR, PIK3C2B, PIK3CB, PIK3R4, PISRT1, PKP1, PLEKHA6, PLXNA1, PLXNA2, PLXND1, PM20D1, PODXL2, POLQ, PPFIA4, PPP1R12B, PPP1R15B, PPP2R3A, PPP2R5A, PRELP, PROX1, PRR23A, PRR23B, PRR23C, PRSS38, PSEN2, PTCH1, PTPDC1, PTPLB, PTPN14, PTPN7, PTPRC, PTPRVP, PYCR2, RAB3GAP2, RAB43, RAB4A, RAB6B, RAB7A, RAB7L1, RABIF, RASSF5, RBBP5, RBM34, RBP1, RBP2, RCOR3, RD3, REN, RHO, RHOU, RNF187, RNPEP, RNU5F-1, ROPN1, ROPN1B, ROR2, RPL32P3, RPN1, RPS10P7, RPS6KC1, RRP15, RUVBL1, RYK, RYR2, S1PR3, SDE2, SEC22A, SEC61A1, SEC61B, SECISBP2, SEMA4D, SEMA5B, SERTAD4, SERTAD4-AS1, SHC3, SHISA4, SIPA1L2, SLC12A8, SLC15A2, SLC26A9, SLC30A1, SLC30A10, SLC35D2, SLC35F3, SLC35G2, SLC41A1, SLC41A3, SLC45A3, SLCO2A1, SMYD2, SNAP47, SNORA14B, SNORA16B, SNORA24, SNORA36B, SNORA58, SNORA77, SNORA7B, SNORA84, SNORD112, SNRPD2P2, SNRPE, SNX4, SOX13, SOX14, SPATA17, SPATA31C1, SPATA31C2, SPATA31E1, SPHAR, SPIN1, SPRTN, SPTLC1, SRGAP2, SRGAP2C, SRP9, SRPRB, STAG1, STX17, STXBP5L, SUSD3, SUSD4, SYK, SYT14, SYT2, TAF1A, TAF5L, TARBP1, TATDN3, TBC1D2, TBCE, TDRD7, TEX10, TF, TGFB2, TGFBR1, TIMM17A, TLR5, TMCC1, TMCC2, TMEM108, TMEM183A, TMEM206, TMEM63A, TMEM81, TMEM9, TMOD1, TNNI1, TNNT2, TOMM20, TOPBP1, TP53BP2, TPRA1, TRAF3IP3, TRAF5, TRH, TRIM11, TRIM14, TRIM17, TRIM42,SLC35F3, TRIM67, TRNA_Cys, TRNA_Glu, TRNA_Lys, TRNA_Pseudo, TRNA_Thr, TSNAX, TSNAX-DISC1, TSTD2, TTC13, TXNRD3, TXNRD3NB, U4, U42379, U6, U6atac, U7, UBA5, UBE2T, UMPS, UNQ2790, UNQ6494, URB2, UROC1, USH2A, VASH2, WDR26, WDR5B, WNK2, WNT3A, WNT9A, XPA, YOD1, ZBED6, ZC3H11A, ZNF148, ZNF169, ZNF281, ZNF322, ZNF367, ZNF484, ZNF510, ZNF678, ZNF782, ZNF847P,5S_rRNA, ZXDC, mir-29b-2
DNA repair	*ABL1, ACTB, ACTL6A, ACTR5, ACTR8, ALKBH2, ALKBH3, ALKBH4, APBB1, APE1, APE2, APEX1, APEX2, APEX3, APEX4, APITD1-CORT, APTX, AQR, ASCC1, ASCC2, ASCC3, ATR, ATRIP, BABAM1, BABAM2, BARD2, BAZ1B, BIVM-ERCC5, BIVM-ERCC6, BRCA3, BRCC3, BRCC4, BRE, BRIP2, CCNA1, CCNA2, CCNB1, CCNH, CDC25C, CDK1, CDK2, CDK3, CDK7, CDK8, CEN2, CENPS, CENPX, CETN2, CETN3, CHD1L, CHEK1, CLSPN, COPS2, COPS3, COPS4, COPS5, COPS6, COPS7A, COPS7B, COPS8, CSA, CSB, CUL4A, CUL4B, CtIP, DCLRE1A, DCLRE1B, DCLRE1C, DDB1, DDB1-XPE, DDB3, DDB4, DNA, DNA-PKc, DNA2, DNTT, DTL, ELL, EME1, EME2, EME3, EME4, EP300, ERCC1, ERCC6, ERCC8, EYA1, EYA2, EYA3, EYA4, Exo2, FAAP100, FAAP101, FAAP20, FAAP24, FAAP25, FAM175A, FAN2, FANCD3, FEN1, FEN2, FSBP, GEN1, GEN2, GPS1, GTF2H1, GTF2H2, GTF2H2C, GTF2H2C_2, GTF2H2C_3, GTF2H3, GTF2H4, GTF2H5, GTF2H6, GTF2H7, GTF2H8, H2AFX, HERC2, HES1, HES2, HIST1H2BA, HIST1H2BB, HIST1H2BC, HIST1H2BD, HIST1H2BE, HIST1H2BF, HIST1H2BG, HIST1H2BH, HIST1H2BI, HIST1H2BJ, HIST1H2BK, HIST1H2BL, HIST1H2BM, HIST1H2BN, HIST1H2BO, HIST1H4A, HIST1H4B, HIST1H4C, HIST1H4D, HIST1H4E, HIST1H4F, HIST1H4H, HIST1H4I, HIST1H4J, HIST1H4K, HIST1H4L, HIST2H2BE, HIST2H4A, HIST2H4B, HIST3H2BB, HIST3H3, HIST4H4, HMGB1, HMGB2, HMGN1, HUS1, INO80, INO80B, INO80C, INO80D, INO80E, ISG15, ISY1, KAT5, KDM4A, KDM4B, KIAA0101, KPNA2, Ku70, Ku80, LIG1, LIG2, LIG3, LIG4, LIG5, LIG6, LIG7, LOC102724334, LOC105369236, MAD2L2, MAPK8, MBD4, MBD5, MCRS1, MCRS2, MDC1, MGMT, MHF1, MLH2, MLH4, MNAT1, MNAT2, MPG, MRE11, MRE11A, MRE12, MUS81, NEIL1, NEIL2, NEIL3, NEIL4, NEIL5, NEIL6, NFRKB, NHEJ1, NHEJ2, NPLOC4, NTHL2, Nbs1, Nbs2, OGG1, OGG2, PALB3, PARG, PARP, PARP1, PARP2, PARP3, PARP4, PARP5, PARP6, PARP7, PARP8, PAXIP1, PCNA, PCNA-RFC, PIAS1, PIAS3, PIAS4, PMS3, PNKP, POLA1, POLA2, POLB, POLD2, POLD3, POLD4, POLD5, POLD6, POLD7, POLD8, POLE2, POLE3, POLE4, POLE5, POLE6, POLE7, POLH, POLI, POLK, POLL, POLM, POLN, POLQ, POLR1A, POLR2A, POLR2B, POLR2C, POLR2D, POLR2E, POLR2F, POLR2G, POLR2H, POLR2I, POLR2J, POLR2K, POLR2L, PPIE, PPP4C, PPP4R2, PPP5C, PRKDC, PRPF19, RAD1, RAD17, RAD18, RAD23A, RAD23B, RAD50, RAD51, RAD51AP1, RAD51B, RAD52, RAD53, RAD54, RAD54B, RAD54L, RAD9A, RAD9B, RBBP8, RBBP9, RBX1, RBX2, RCHY1, REV1, REV2, REV3L, RFC1, RFC10, RFC2, RFC3, RFC4, RFC5, RFC6, RFC7, RFC8, RFC9, RHNO1, RIF1, RMI1, RMI2, RMI3, RMI4, RNF111, RNF168, RNF4, RNF8, RPA, RPA1, RPA2, RPA3, RPA4, RPA5, RPA6, RPA7, RPA8, RPS27A, RTEL1, RTEL2, RUVBL1, RUVBL2, SFN, SHFM1, SHFM2, SIRT6, SIRT7, SLX1, SLX1A, SLX1B, SMARCA5, SMARCA6, SMUG1, SMUG2, SPIDR, SPRTN, SSBP1, SSBP2, SUMO1, SUMO2, SUMO3, SYCP3, SYCP4, TCEA1, TDG, TDP1, TDP2, TELO2, TELO3, TFIIH, TFPT, TIMELESS, TIPIN, TOP3A, TOP3B, TOPBP1, TOPBP2, TP53BP1, TRIM25, UBA52, UBA7, UBB, UBC, UBE2B, UBE2I, UBE2L6, UBE2N, UBE2T, UBE2V2, UFD1L, UIMC1, UIMC2, UNG, USP1, USP10, USP2, USP43, USP45, USP7, UV-DDB, UVSSA, VCP, WDR48, WDR49, WEE1, WEE2, WHSC1, XAB2, XLF, XPB, XPD, XPF, XPG, XRCC1, XRCC2, XRCC3, XRCC4, XRCC5, XRCC6, YWHAB, YWHAE, YWHAG, YWHAH, YWHAQ, YWHAZ, YY1, Yen1, ZBTB32, ZBTB33, ZNF830*
Pathways in cancer	*ACVR1, ACVR1B, ACVR1C, ACVR2A, ACVR2B, ACVRL1, ADCY1, ADCY2, ADCY3, ADCY4, ADCY5, ADCY6, ADCY7, ADCY8, ADCY9, AGTR1, AKT1, AKT2, AKT3, AMH, AMHR2, ANKRD6, APC2, APC3, APPL1, APPL2, AR, ARAF, ARHGEF1, ARHGEF11, ARHGEF12, ARRB1, ARRB2, ATF1, ATF2, ATF4, AXIN1, AXIN3, AXIN4, BAD, BAMBI, BCL2, BCL2L1, BCL9, BCR, BDKRB1, BDKRB2, BIRC2, BIRC3, BIRC5, BIRC6, BIRC7, BIRC8, BMP2, BMP4, BMP5, BMP6, BMP7, BMP8A, BMP8B, BMPR1B, BMPR2, BRAF, BRD7, BTRC, CACYBP, CAMK2A, CAMK2B, CAMK2D, CAMK2G, CASP15, CASP21, CASP3, CASP9, CBL, CBLB, CBLC, CCDC6, CCND1, CCND2, CCND3, CCNE1, CCNE2, CDC42, CDH2, CDK6, CDKN1A, CDKN1B, CDKN2B, CEBPA, CER1, CHD8, CHRD, CHUK, CKS1B, CKS2, COL4A1, COL4A2, COL4A3, COL4A4, COL4A5, COL4A6, CREB1, CREB3L1, CREBBP, CRK, CRKL, CRYBB2, CSF1R, CSF2RA, CSF3R, CSNK1A1, CSNK1A1L, CSNK1D, CSNK1E, CSNK2A1, CSNK2A2, CSNK2A3, CSNK2B, CTBP1, CTBP2, CTNNA1, CTNNA2, CTNNA3, CTNNB1, CTNNB2, CTNNBIP1, CUL1, CXCL12, CXCL8, CXCR4, CXXC4, DAAM1, DAAM2, DAB2, DAPK1, DAPK2, DAPK3, DAXX, DCC, DCN, DKK1, DKK2, DKK4, DLG1, DLG2, DLG4, DVL1, DVL2, DVL3, E2F1, E2F2, E2F3, E2F4, E2F5, EDNRA, EDNRB, EGF, ERBB2, ESR1, ESR2, F2R, F2RL3, FBXW11, FGF1, FGF10, FGF11, FGF12, FGF13, FGF14, FGF16, FGF17, FGF18, FGF19, FGF2, FGF20, FGF21, FGF22, FGF23, FGF3, FGF4, FGF5, FGF6, FGF7, FGF8, FGF9, FGFR1, FGFR2, FGFR3, FHL2, FLT3, FLT3LG, FN1, FOS, FOSB, FOSL1, FOXH1, FOXO1, FOXO3, FRAT1, FRAT2, FST, FZD1, FZD10, FZD2, FZD3, FZD4, FZD5, FZD6, FZD7, FZD8, FZD9, GDF5, GDF6, GDF7, GLI3, GNA11, GNA12, GNA13, GNAI1, GNAI2, GNAI3, GNAQ, GNAS, GNB1, GNB2, GNB3, GNB4, GNB5, GNG10, GNG11, GNG12, GNG13, GNG2, GNG3,*

	GNG4, GNG5, GNG7, GNG8, GNGT1, GNGT2, GPC4, GRB2, GRB2, GSK3B, HDAC1, HDAC2, HGF, HIPK2, HNF4A, HNF4G, ID1, ID2, ID3, ID4, IFNG, IGF1, IGF1R, IKBKB, IKBKG, IL6, INHBA, INHBB, INHBC, INHBE, INVS, ITGA2, ITGA2B, ITGA3, ITGA6, ITGAV, ITGB1, JAK1, JUN, JUNB, JUND, JUP, KITLG, KRAS, LAMA1, LAMA2, LAMA3, LAMA4, LAMA5, LAMB1, LAMB2, LAMB3, LAMB4, LAMC1, LAMC2, LAMC3, LEF1, LEF2, LEFTY1, LEFTY2, LOC400927-CSNK1E, LOC644172, LPAR1, LPAR2, LPAR3, LPAR4, LPAR5, LPAR6, LRP1, LRP5, LTBP1, MAGI3, MAP1B, MAP2K1, MAP2K2, MAP2K3, MAP2K4, MAP2K6, MAP3K7, MAPK1, MAPK10, MAPK11, MAPK12, MAPK13, MAPK14, MAPK3, MAPK8IP1, MAPK9, MARK2, MDM2, MECOM, MEF2C, MET, MINOS1-NBL1, MIR3661, MITF, MMP1, MMP2, MMP7, MMP9, MTOR, MYB, MYC, MYOD1, NBL1, NCOA4, NFATC1, NFATC2, NFATC3, NFATC4, NFKB1, NFKB2, NFKBIA, NKD1, NKD2, NKX3-1, NLK, NODAL, NOG, NOS2, NOTUM, NR3C1, NR5A1, NRAS, NTRK1, PAK2, PAX2, PAX8, PDGFA, PDGFB, PDGFRA, PDGFRB, PDPK1, PGF, PIAS2, PIK3C2B, PIK3CA, PIK3CB, PIK3CD, PIK3CG, PIK3R1, PIK3R2, PIK3R3, PIK3R5, PIN1, PITX2, PLCB1, PLCB2, PLCB3, PLCB4, PLCG1, PLCG2, PLEKHG5, PML, PORCN, PPARD, PPARG, PPP2CA, PPP2CB, PPP2R1A, PPP2R1B, PPP3CA, PPP3CB, PPP3CC, PPP3R1, PPP3R2, PRICKLE1, PRICKLE2, PRKACA, PRKACB, PRKACG, PRKCA, PRKCB, PRKCG, PSEN1, PTEN, PTGER1, PTGER2, PTGER3, PTGER4, PTGS2, PTK2, RAC1, RAC2, RAC3, RAF1, RALA, RALB, RALBP1, RALGDS, RARA, RARB, RASGRP1, RASGRP2, RASGRP3, RASGRP4, RASSF1, RASSF5, RBL1, REL, RELA, RELB, RHOA, ROCK1, ROCK2, ROR2, RPS6KB1, RPS6KB2, RUNX1T1, RUNX2, RUNX3, RXRA, RXRB, RXRG, SALL1, SENP2, SERPINF1, SFRP1, SFRP2, SFRP4, SFRP5, SHC1, SIAH1, SKP1, SKP2, SMAD1, SMAD2, SMAD3, SMAD5, SMAD6, SMAD7, SMURF1, SMURF2, SOS1, SOS2, SOST, SOX1, SOX17, SOX9, SP1, SPI1, STAT1, STAT3, STAT5A, STAT5B, STK4, SUMO2, TAX1BP3, TBL1X, TBL1XR1, TBL1Y, TBP, TCF3, TCF4, TCF7, TCF7L1, TCF7L2, TCF7L3, TCF7L4, TCF8, TFAP2A, TFDP1, TFG, TGFA, TGFB1, TGFB2, TGFB3, TGFBR1, TGFBR2, TGFBR3, TGIF1, TGIF2, THBS1, TNF, TPM3, TPR, TRAF1, TRAF2, TRAF3, TRAF4, TRAF5, TRAF6, VANGL1, VANGL2, VEGFA, VEGFB, VEGFC, VEGFD, WIF1, WNT1, WNT10A, WNT10B, WNT11, WNT16, WNT2, WNT21, WNT26, WNT2B, WNT3, WNT3A, WNT4, WNT5, WNT5A, WNT5B, WNT6, WNT7, WNT7A, WNT7B, WNT8A, WNT8B, WNT9A, WNT9B, XIAP, ZBTB16, ZFYVE16, ZFYVE9

Table S3. List of rare variants in candidate genes from the WES germline analyses. Variants with MAF gnomAD < 1% were selected and filtered based on pathogenicity prediction criteria. All variants are paternally-inherited. *CADD; DANN, GERP:* in silico predictors of pathogenicity.

Chromosome	Start	End	Reference Allele	Alternative Allele	Genotype	Gene	Variant	Location	Automated InterVar
1	210012345	210012345	T	C	het	*DIEXF*	NM_014388:c.1154T>C, p.(I385T)	exonic	Uncertain significance
3	133099265	133099265	C	A	het	*TMEM108*	NM_023943:c.710C>A, p.(P237H)	exonic	Uncertain significance
**9**	**97876996**	**97876996**	**C**	**T**	**het**	***FANCC***	**NM_000136:c.1073-4G>A**	**splicing/intronic**	**-**
**12**	**102813337**	**102813337**	**C**	**T**	**het**	***IGF1***	**NM_000618:c.352G>A, p.(A118T)**	**exonic**	**Likely pathogenic**

Table S3. Continued.

Chromosome	Start	End	ClinVar	SNP ID	gnomAD NFE	CADD phred	DANN score	GERP++	Interpro_domain
1	210012345	210012345	-	rs751920227	0	17.54	0.986	3.54	P-loop containing nucleoside triphosphate hydrolase
3	133099265	133099265	-	-	0	25.3	0.994	3.27	-
**9**	**97876996**	**97876996**	**Conflicting interpretations of pathogenicity**	**rs147695697**	**0**	**-**	**-**	**-**	**-**
**12**	**102813337**	**102813337**	**Uncertain significance**	**rs151098426**	**0.0015**	**24.1**	**0.998**	**5.85**	**Insulin, conserved site|Insulin-like**

Table S4. Candidate variant prioritization with eDiVa. Table with the most likely rare functional variants as produced by the eDiVa software (http://ediva.crg.eu). Variants are ranked by pathogenicity prediction. Variants were produced for paternally inherited dominant, dominant de novo or recessive models of inheritance and filtered according to the quality criteria described in Material and Methods. Of note, no hits were found for the recessive or de novo changes that withstood our QC criteria, and hence all presented variants are paternally-inherited.

Chromosome	Position	Reference Allele	Alternative Allele	Genotype	Gene	Variant	Location	Rank
**2**	**120639370**	**A**	**G**	**Heterozygous**	***PTPN4***	**NM_002830:c.A377G, p.(Y126C)**	**exonic**	**1**
20	46313306	C	T	Heterozygous	*SULF2*	NM_001161841:c.G757A, p.(A253T)	exonic	0.985
2	160284496	T	C	Heterozygous	*BAZ2B*	NM_013450:c.A2422G, p.(K808E)	exonic	0.866
6	18143955	C	G	Heterozygous	*TPMT*	NM_000367:c.G238C, p.(A80P)	exonic	0.705
1	40124935	C	T	Heterozygous	*NT5C1A*	NM_032526:c.G965A, p.(R322H)	exonic	0.57
11	102477309	C	T	Heterozygous	*MMP20*	NM_004771:c.G910A, p.(A304T)	exonic	0.43
**1**	**11852412**	**G**	**A**	**Heterozygous**	***MTHFR***	**NM_005957:c.C1555T, p.(R519C)**	**exonic**	**0.391**
10	49440275	C	T	Heterozygous	*FRMPD2*	NM_001018071:c.G1051A, p.(G351R)	exonic	0.355
9	72755177	C	T	Heterozygous	*MAMDC2*	NM_153267:c.C1111T:p.R371W	exonic	0.33
2	38916968	T	C	Heterozygous	*GALM*	NM_138801:c.T566C, p.(I189T)	exonic	0.303
10	1038477	G	A	Heterozygous	*GTPBP4*	NM_012341:c.G97A, p.(V33I)	exonic	0.287
4	129208629	T	C	Heterozygous	*PGRMC2*	NM_006320:c.A389G, p.(E130G)	exonic	0.285
6	39552692	T	G	Heterozygous	*KIF6*	NM_001289020:c.A1059C, p.(E353D)	exonic	0.253
16	29994927	G	A	Heterozygous	*TAOK2*	NM_001252043:c.G1364A, p.(R455H)	exonic	0.219
10	102740358	G	A	Heterozygous	*SEMA4G*	NM_001203244:c.G1375A:p.V459I	exonic	0.2
22	31836842	G	A	Heterozygous	*EIF4ENIF1*	NM_001164502:c.C2042T, p.(P681L)	exonic	0.197
7	117282582	G	A	Heterozygous	*CFTR*	NM_000492:c.G3808A, p.(D1270N)	exonic	0.17
1	24989159	T	A	Heterozygous	*SRRM1*	NM_001303448:c.T1486A, p.(S496T)	exonic	0.161
7	117149143	C	T	Heterozygous	*CFTR*	NM_000492:c.C220T, p.(R74W)	exonic	0.12
**12**	**102813337**	**C**	**T**	**Heterozygous**	***IGF1***	**NM_000618:c.G352A, p.(A118T)**	**exonic**	**0.069**

Table S4. Continued.

Chromosome	Placental Mammal PhyloP	SNP ID	MAF gnomAD NFE	GERP++	CADD	Inheritance
**2**	**2.176**	**NA**	**0**	**5.41**	**6.132**	**dominant**
20	2.401	NA	0	4.62	6.201	dominant
2	2.26	NA	0	5.92	5.594	dominant
6	2.675	rs1800462	0.002	5.38	6.372	dominant
1	2.624	rs190344471	0.0004	5.26	7.520	dominant
11	2.835	rs148818720	0.002	5.45	6.347	dominant
**1**	**2.426**	**rs45496998**	**0.0003**	**5.18**	**7.179**	**dominant**
10	1.299	rs116143480	0.007	5.44	6.185	dominant
9	2.865	rs148444015	0.0009	6.02	7.857	dominant
2	2.096	rs200292259	0.002	4.8	5.800	dominant
10	2.796	rs150687511	0.0001	5.59	5.199	dominant
4	1.831	rs149247614	0.001	4.06	4.735	dominant
6	2.12	rs150520804	0.00003	5.29	6.163	dominant
16	1.29	NA	NA	5.47	5.912	dominant
10	1.108	rs144844358	0.0001	5.02	3.723	dominant
22	2.763	rs139184009	0.0006	5.89	4.837	dominant
7	2.568	rs11971167	0.0001	5.2	7.224	dominant
1	2.21	rs41268775	0.006	5.51	3.092	dominant
7	2.681	rs115545701	0.0003	5.68	4.341	dominant
**12**	**2.768**	**rs151098426**	**0.001**	**5.85**	**4.354**	**dominant**

Table S5. Modifier genes in LS. Here, we represent the genes that have been proposed to modify CRC risk in a LS setting. We have included all the genes that have to date been described as modifiers, regardless of ambiguous reports. An extensive report on the evidence for each gene can be found in reference [5].

Category	Gene	Reference
Xenobiotic clearance and micronutrient metabolism	*NAT1*	[6]
	NAT2	[6]
	GST	[6]
	CYP1A1	[6]
	CYP17A1	[7]
Cell cycle control	*TP53*	[8]
	AURKA	[9]
	CCND1	[10]
	ATM	[11]
	KIF20A	[12]
	CDC25C	[12]
	KDM3B/FAM53C	[12]
DNA repair	*MSH3*	[13]
	OGG1	[13]
	XRCC1	[13]
	XRCC2	[13]
	XRCC3	[13]
	BRCA2	[13]
	Lig4	[13]
	TERT	[14]
Immunological function	*TNF-alpha*	[15]
	IL6	[15]
	IL8	[15]
	NFKB1	[15]
	PPARG	[15]
Growth factors	*IGF1*	[16]
Other	*MTHFR*	[17]
	HFE	[18]
	DNMT3B	[19]
	8q23.3-rs16892766	[20]
	11q23.1-rs3802842	[20]
	SMAD7	[21]
	APC	[22]
	CHEK2	[23]

Table S6. List of variants found in the described Lynch syndrome modifier genes. The list includes all changes found in the genes gathered in Supplementary Table S6 that withstood QC as described in the materials and methods. All variants are paternally inherited except: *mother is wild-type homozygous, father is homozygous for the variant allele; **mother is heterozygous, father is homozygous for the variant allele.

Chromosome	Start	End	Reference Allele	Alternative Allele	Genotype	Gene	Variant	Location
1	110233147	110233147	C	T	homozygous	*GSTM1*	NM_000561:c.528C>T, p.(D176D) *	exonic
**1**	**11852412**	**11852412**	**G**	**A**	**heterozygous**	***MTHFR***	**NM_005957:c.1555C>T, p.(R519C)**	**exonic**
5	137771386	137771386	C	G	heterozygous	*KDM3B*	NM_016604:c.5283C>G, p.(S1761S)	exonic
6	26091336	26091336	T	C	homozygous	*HFE*	NM_000410.3:c.340+4T>C**	splicing;intronic
10	104596924	104596924	C	A	heterozygous	*CYP17A1*	NM_000102:c.195G>T, p.(S65S)	exonic
10	104596981	104596981	G	A	heterozygous	*CYP17A1*	NM_000102:c.138C>T, p.(H46H)	exonic
**11**	**69462910**	**69462910**	**G**	**A**	**heterozygous**	***CCND1***	**NM_053056:c.723G>A, p.(P241P)**	**exonic**
**12**	**102813337**	**102813337**	**C**	**T**	**heterozygous**	***IGF1***	**NM_000618:c.352G>A, p.(A118T)**	**exonic**

Table S7. GWAS loci used for PRS score. List of low-penetrance risk alleles for CRC with their corresponding effect sizes [24–38].

Risk locus	SNP ID	Odds Ratio	Risk allele	Reference
1p36.12	rs72647484	1.13	T	[24]
1q25.3	rs10911251	1.08	A	[25]
1q41	rs6691170	1.08	T	[26]
2q35	rs992157	1.08	A	[27]
3q26.2	rs10936599	1.07	C	[26]
5q31.1	rs647161	1.04	A	[28]
6p21.1	rs4711689	1.03	A	[29]
6p21.2	rs1321311	1.09	A	[30]
8q23.3	rs16892766	1.26	C	[31]
8q24.21	rs6983267	1.19	G	[32]
10p14	rs10795668	1.11	G	[31]
10q22.3	rs704017	1.1	G	[33]
10q24.2	rs1035209	1.11	T	[25]
10q24.32	rs4919687	1.02	G	[29]
10q25.2	rs12241008	1.11	C	[34]
10q25.2	rs11196172	1.08	A	[33]
11q12.2	rs174537	1.05	G	[33]
11q13.4	rs3824999	1.09	G	[30]
11q23.1	rs3802842	1.15	C	[35]
12p13.31	rs10849432	1.14	T	[33]
12p13.32	rs10774214	1.04	T	[28]
12p13.32	rs3217810	1.11	T	[25]
12q13.13	rs11169552	1.06	C	[26]
12q24.12	rs3184504	1.09	C	[36]
14q22.2	rs4444235	1.08	C	[37]
14q22.2	rs1957636	1.06	A	[31]
15q13.3	rs11632715	1.07	A	[31]
16q22.1	rs9929218	1.06	G	[37]
18q21.1	rs4939827	1.2	T	[38]
19q13.11	rs10411210	1.14	C	[37]
19q13.2	rs1800469	1.08	G	[33]
20p12.3	rs961253	1.11	A	[37]
20p12.3	rs2423279	1.08	C	[28]
20p12.3	rs4813802	1.09	G	[31]
20q13.13	rs6066825	1.1	C	[36]
20q13.33	rs4925386	1.16	C	[26]
Xp22.2	rs5934683	1.08	T	[30]

Supplementary Figures

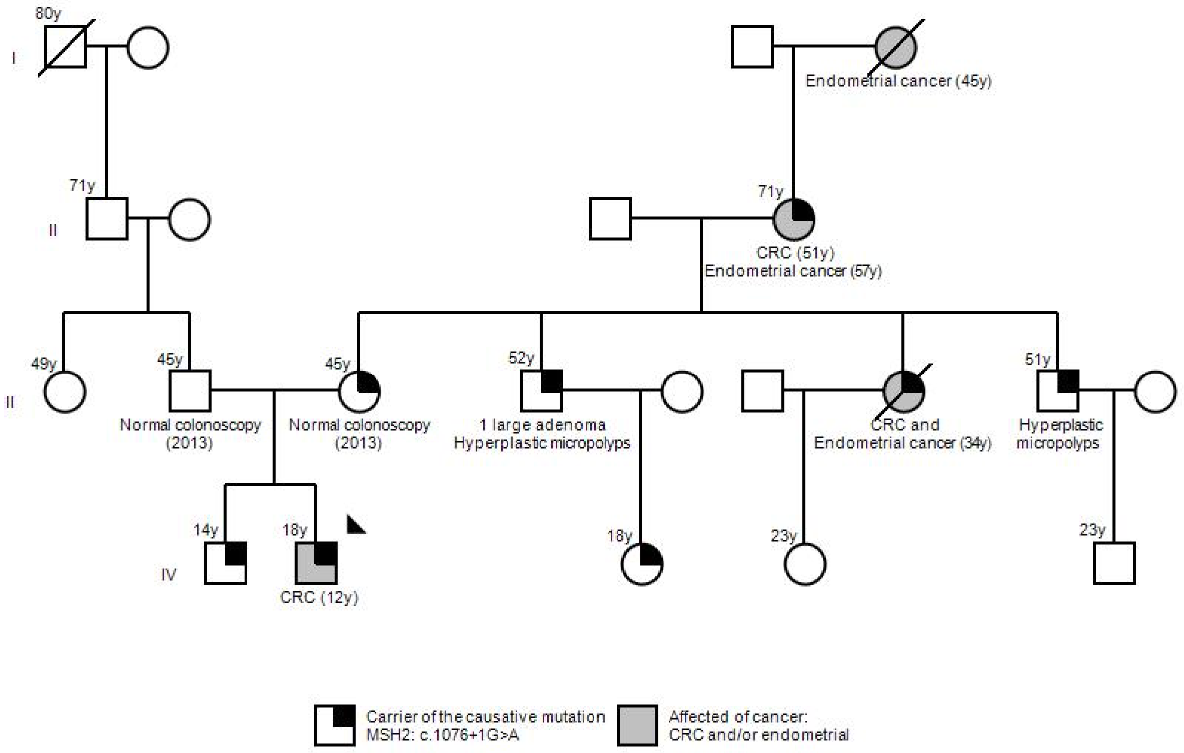

Figure S1. Family pedigree. Pedigree of the patient within the LS family showing mutation carriers and cancers.

A

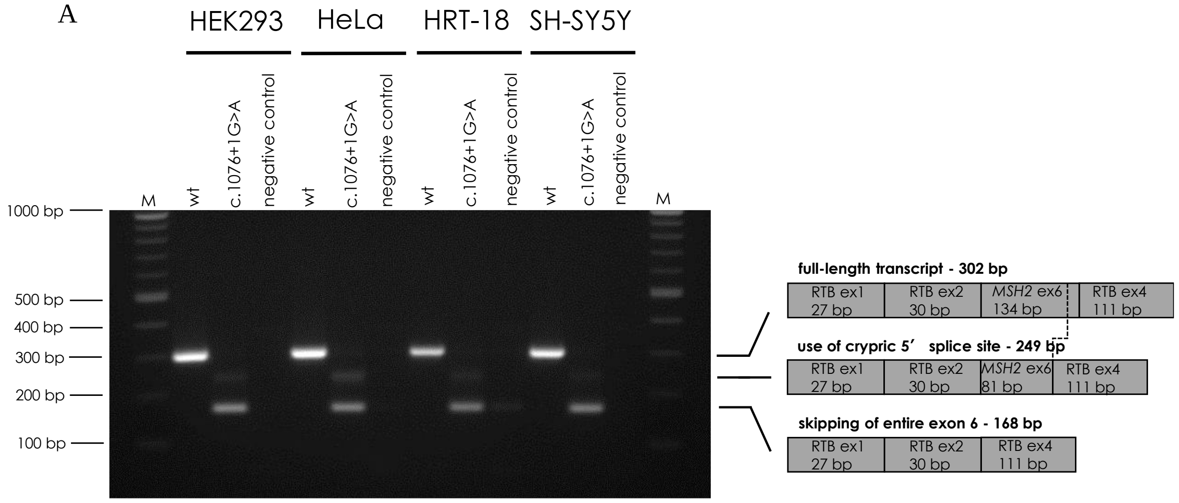

Figure S2. *Cont.*

B

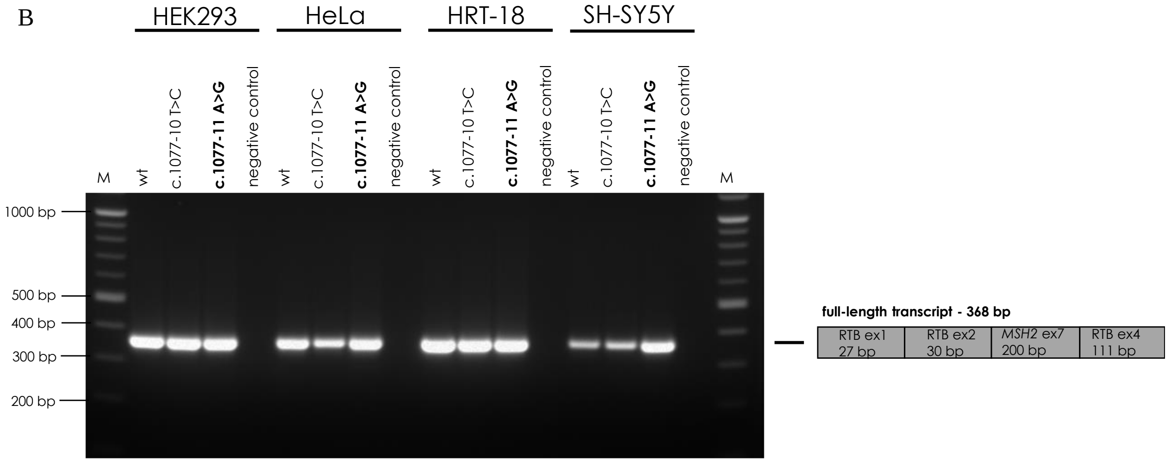

Figure S2. Minigene experiments. Design and results of the minigene experiments performed to assess the spliceogenic potential of the MSH2 c.1077-11A>G variant as compared to the known c.1076+1G>A pathogenic change and the c.1077-10C>T polymorphism. (**A**) electrophoresis gel showing the fragments produced with the known pathogenic variant, which includes a 168 bp fragment consistent with exon 6 skipping; (**B**) electrophoresis gel for the minigene experiments for variant c.1077-11G>A showing no evidence of splicing.

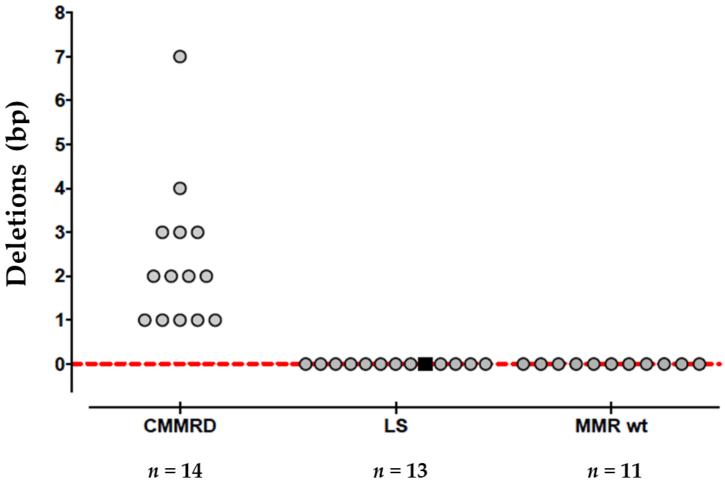

Figure S3. Ex vivo MSI assays. We depict the evMSI for our patient (black square) amongst other with confirmed CMMRD/LS diagnosis, or healthy controls. We can observe that the deletion sizes (in base pairs [bp]) are expressed as the sum of the deletions for the 3 microsatellite markers (NR27, NR21, and BAT26). The cut-off value used to define a lymphoblastoid cell line as positive for evMSI was set at 1 bp deletion (red dotted line). We observe that our patient is included in the LS-to-control range, thereby exluding the presence of germline MMR deficiency (adapted from Bodo et al. 2015 [39]).

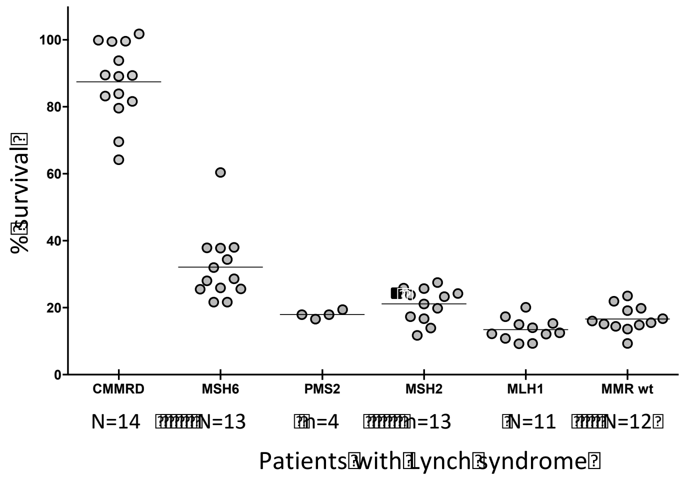

Figure S4. Methylation tolerance assays. Tolerance (measured as % survival) of immortalized lymphoblasts derived from the patient (black square) and others (grey circles) with LS/CMMRD phenotype and healthy controls (MMR wt). We can observe that our patient presents a tolerance range lower than 40%, which is similar to other LS patients and controls (adapted from Bodo et al. 2015 [39]).

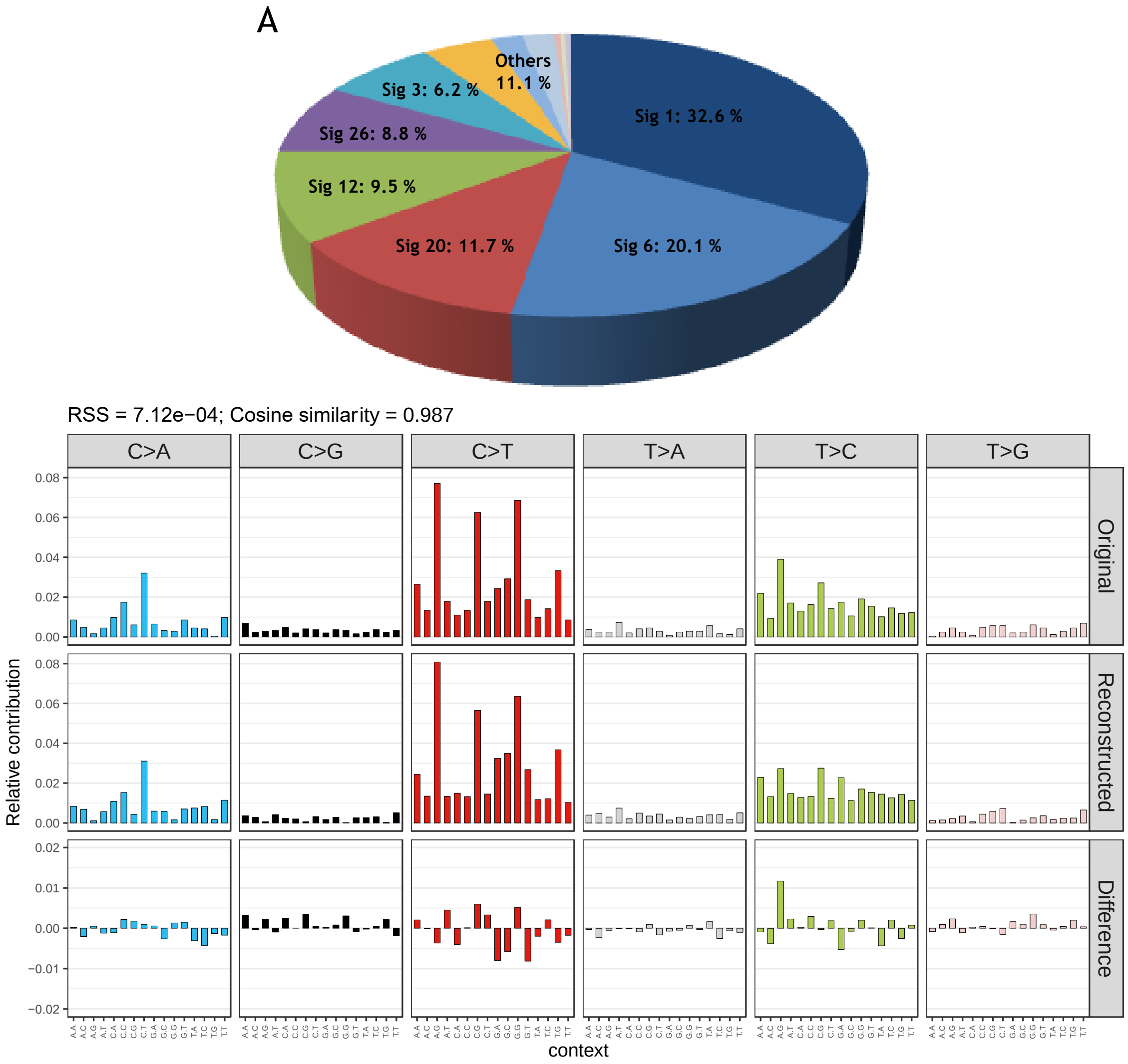

Figure S5. *Cont.*

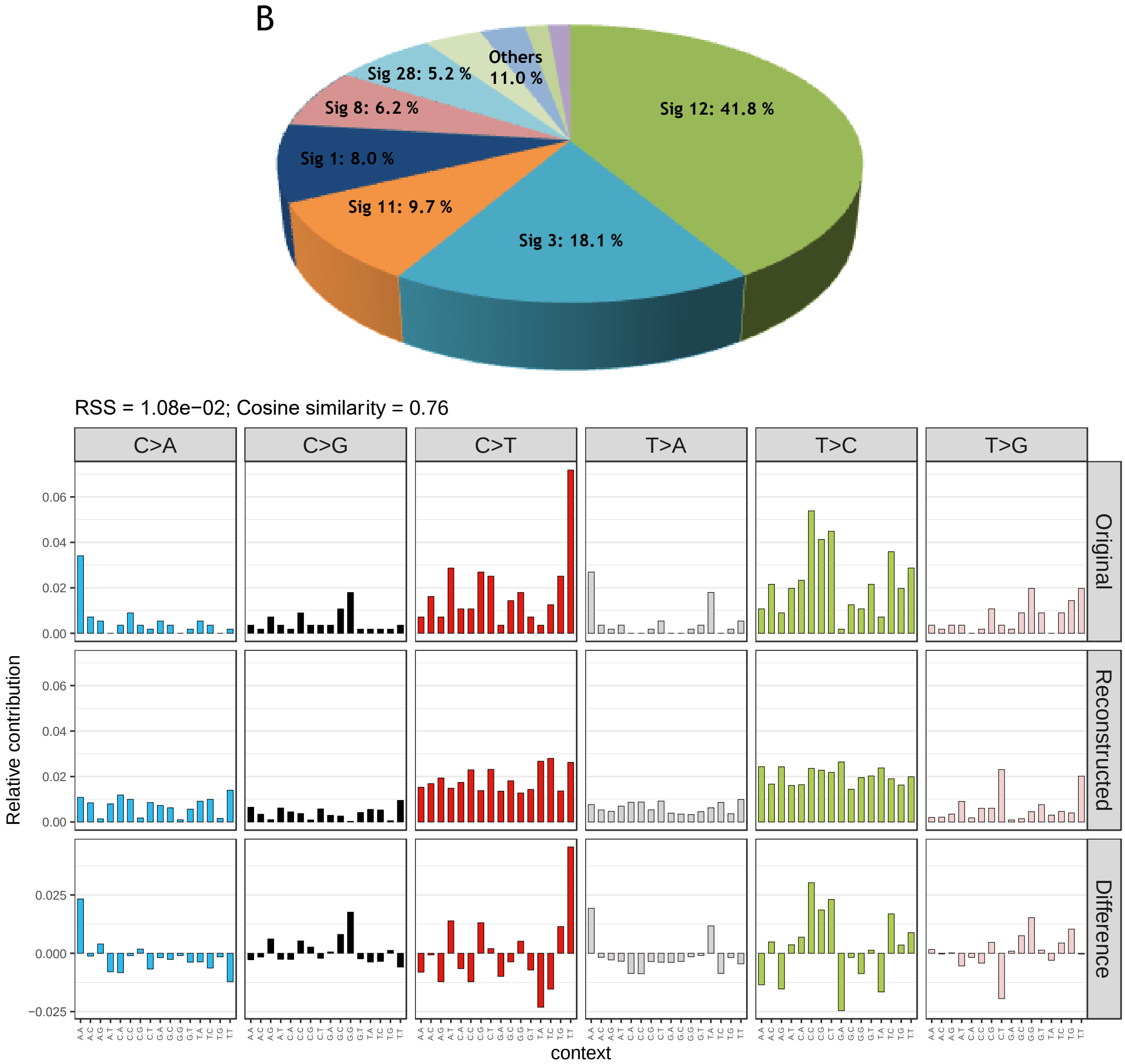

Figure S5. Mutational signature of the somatic tissues and 96-spectrum mutation plots. A: tumour; we observe a predominance of signatures related to impaired repair (signatures 3, 6, 20, 26) and MSI (signature 1), while the spectrum is dominated by C>T transitions; B: normal adjacent mucosa. We do not observe the C>T transitions typical of the spontaneous deamination events suffered in paraffin-embedded tissues. In turn, we observe a great contribution of T>C transitions (signature 12), which has been related to an undefined signature features in liver cancers.

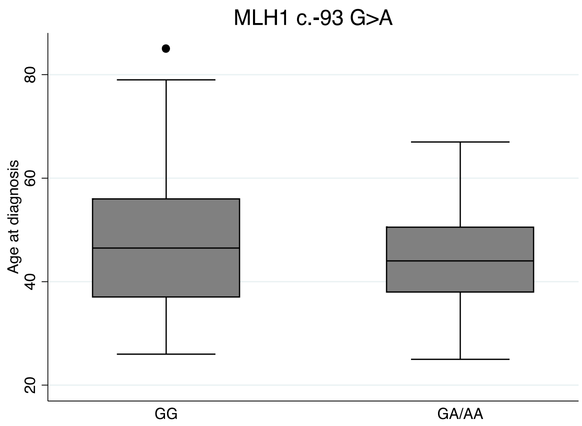

Figure S6. Comparison of age of onset relative to the MLH1 c-93 G>A variant genotype. We compared the average age of onset between the patients with the wild type and risk alleles

following a dominant mode of inheritance. The average ages for these groups were μ_{GG} = 47.66 (range (26–85), *n* = 74) and $\mu_{GA/AA}$ = 44.89 (range (25–67, *n* = 46), respectively. Although non-significant, there seems to be a trend towards younger ages at diagnosis for patients carrying the risk A allele of this SNP.

Reference

1. Kanehisa, M.; Furumichi M.; Tanabe M.; Sato Y.; Morishima.; K. KEGG: New perspectives on genomes, pathways, diseases and drugs. *Nucleic Acids Res.* **2017**, *45*, D353–D361.
2. Kemp, Z.; Carvajal-Carmona, L.; Spain, S.; Barclay, E.; Gorman, M.; Martin.; L.; Jaeger, E.; Brooks, N.; Bishop, D.T.; Thomas, H.; et al. Evidence for a colorectal cancer susceptibility locus on chromosome 3q21-q24 from a high-density SNP genome-wide linkage scan. *Hum. Mol. Genet.* **2006**, *15*, 2903–2910.
3. Middeldorp, A.; Jagmohan-Changur, S.C.; van der Klift, H.M.; van Puijenbroek, M.; Houwing-Duistermaat, J.J.; Webb, E.; Houlston, R.; Tops, C.; Vasen, H.F.; Devilee, P.; et al. Comprehensive genetic analysis of seven large families with mismatch repair proficient colorectal cancer. *Genes Chromosomes Cancer* **2010**, *49*, 539–548.
4. Wiesner, G.L.; Daley, D.; Lewis, S.; Ticknor, C.; Platzer, P.; Lutterbaugh, J.; MacMillen, M.; Baliner, B.; Willis, J.; Elston, R.C.; et al. A subset of familial colorectal neoplasia kindreds linked to chromosome 9q22.2-31.2. *Proc. Natl. Acad. Sci. USA.* **2003**, *100*, 12961–12965.
5. Talseth-Palmer, B.A.; Wijnen, J.T.; Grice, D.M.; Scott, R.J. Genetic modifiers of cancer risk in Lynch syndrome: A review. *Fam. Cancer* **2013**, *12*, 207–216.
6. Talseth, B.A.; Meldrum, C.; Suchy, J.; Kurzawski, G.; Lubinski, J.; Scott, R.J. Genetic polymorphisms in xenobiotic clearance genes and their influence on disease expression in hereditary nonpolyposis colorectal cancer patients. *Cancer Epidemiol. Biomarkers Prev.* **2006**, *15*, 2307–2310.
7. Campbell, P.T.; Edwards, L.; McLaughlin, J.R.; Green, J.; Younghusband, H.B.; Woods, M.O. Cytochrome P450 17A1 and catechol O-methyltransferase polymorphisms and age at Lynch syndrome colon cancer onset in Newfoundland. *Clin. Cancer Res.* **2007**, *13*, 3783–3788.
8. Jones, J.S.; Chi, X.; Gu, X.; Lynch, P.M.; Amos, C.I.; Frazier, M.L. p53 polymorphism and age of onset of hereditary nonpolyposis colorectal cancer in a Caucasian population. *Clin. Cancer Res.* **2004**, *10*, 5845–5849.
9. Chen, J.; Sen, S.; Amos, C.I.; Wei, C.; Jones, J.S.; Lynch, P.; Frazier, M.L. Association between Aurora-A kinase polymorphisms and age of onset of hereditary nonpolyposis colorectal cancer in a Caucasian population. *Mol. Carcinog.* **2007**, *46*, 249–256.
10. Talseth, B.A.; Ashton, K.A.; Meldrum, C.; Suchy, J.; Kurzawski, G.; Lubinski, J.; Scott, R.J. Aurora-A and Cyclin D1 polymorphisms and the age of onset of colorectal cancer in hereditary nonpolyposis colorectal cancer. *Int. J. Cancer* **2008**, *122*, 1273–1277.
11. Jones, J.S.; Gu, X.; Lynch, P.M.; Rodriguez-Bigas, M.; Amos, C.I.; Frazier, M.L. ATM polymorphism and hereditary nonpolyposis colorectal cancer (HNPCC) age of onset (United States). *Cancer Causes Control* **2005**, *16*, 749–753.
12. Chen, J.; Pande, M.; Huang, Y.J.; Wei, C.; Amos, C.I.; Talseth-Palmer, B.A.; Meldrum, C.J.; Chen, W.V.; Gorlov, I.P.; Lynch, P.M.; et al. Cell cycle-related genes as modifiers of age of onset of colorectal cancer in Lynch syndrome: A large-scale study in non-Hispanic white patients. *Carcinogenesis* **2013**, *34*, 299–306.
13. Reeves, S.G.; Meldrum, C.; Groombridge, C.; Spigelman, A.; Suchy, J.; Kurzawski, G.; Lubinski, J.; Scott, R.J. DNA repair gene polymorphisms and risk of early onset colorectal cancer in Lynch syndrome. *Cancer Epidemiol.* **2012**, *36*, 183–189.
14. Bellido, F.; Guino, E.; Jagmohan-Changur, S.; Segui, N.; Pineda, M.; Navarro, M.; Lázaro, C.; Blanco, I.; Vasen, H.F.; Moreno, V.; et al. Genetic variant in the telomerase gene modifies cancer risk in Lynch syndrome. *Eur. J. Hum. Genet.* **2013**, *21*, 511–516.
15. Landi, S.; Moreno, V.; Gioia-Patricola, L.; Guino, E.; Navarro, M.; de Oca, J.; Capella, G.; Canzian, F.; Bellvitge Colorectal Cancer Study Group. Association of common polymorphisms in inflammatory genes interleukin (IL)6, IL8, tumor necrosis factor alpha, NFKB1, and peroxisome proliferator-activated receptor gamma with colorectal cancer. *Cancer Res.* **2003**, *63*, 3560–3566.
16. Zecevic, M.; Amos, C.I.; Gu, X.; Campos, I.M.; Jones, J.S.; Lynch, P.M.; Rodriguez-Bigas, M.A.; Frazier, M.L. IGF1 gene polymorphism and risk for hereditary nonpolyposis colorectal cancer. *J. Natl. Cancer Inst.* **2006**, *98*, 139–143.

17. Reeves, S.G.; Meldrum, C.; Groombridge, C.; Spigelman, A.D.; Suchy, J.; Kurzawski, G.; Lubinski, J.; McElduff, P.; Scott, R.J. MTHFR 677 C>T and 1298 A>C polymorphisms and the age of onset of colorectal cancer in hereditary nonpolyposis colorectal cancer. *Eur. J. Hum. Genet.* **2009**, *17*, 629–635.
18. Shi, Z.; Johnstone, D.; Talseth-Palmer, B.A.; Evans, T.J.; Spigelman, A.D.; Groombridge, C.; Milward, E.A.; Olynyk, J.K.; Suchy, J.; Kurzawski, G.; et al. Haemochromatosis HFE gene polymorphisms as potential modifiers of hereditary nonpolyposis colorectal cancer risk and onset age. *Int. J. Cancer* **2009**, *125*, 78–83.
19. Jones, J.S.; Amos, C.I.; Pande, M.; Gu, X.; Chen, J.; Campos, I.M.; Wei Q, Rodriguez-Bigas, M.; Lynch, P.M.; Frazier, M.L. DNMT3b polymorphism and hereditary nonpolyposis colorectal cancer age of onset. *Cancer Epidemiol. Biomarkers Prev.* **2006**, *15*, 886–891.
20. Wijnen, J.T.; Brohet, R.M.; van Eijk, R.; Jagmohan-Changur, S.; Middeldorp, A.; Tops, C.M.; et al. Chromosome 8q23.3 and 11q23.1 variants modify colorectal cancer risk in Lynch syndrome. *Gastroenterology* **2009**, *136*, 131–137.
21. Win, A.K.; Hopper, J.L.; Buchanan, D.D.; Young, J.P.; Tenesa, A.; Dowty, J.G.; Giles, G.G.; Goldblatt, J.; Winship, I.; Boussioutas, A.; et al. Are the common genetic variants associated with colorectal cancer risk for DNA mismatch repair gene mutation carriers? *Eur. J. Cancer* **2013**, *49*, 1578–1587.
22. Wimmer, K.; Kratz, C.P.; Vasen, H.F.; Caron, O.; Colas, C.; Entz-Werle, N.; Gerdes, A.M.; Goldberg, Y.; Ilencikova, D.; Muleris, M.; et al. Diagnostic criteria for constitutional mismatch repair deficiency syndrome: Suggestions of the European consortium 'care for CMMRD' (C4CMMRD). *J. Med. Genet.* **2014**, *51*, 355–365.
23. Suchy, J.; Cybulski, C.; Wokolorczyk. D.; Oszurek, O.; Gorski, B.; Debniak, T.; Jakubowska, A.; Gronwald, J.; Huzarski, T.; Byrski, T.; et al. CHEK2 mutations and HNPCC-related colorectal cancer. *Int. J. Cancer* **2010**, *126*, 3005–3009.
24. Al-Tassan, N.A.; Whiffin, N.; Hosking, F.J.; Palles, C.; Farrington, S.M.; Dobbins, S.E.; Harris, R.; Gorman, M.; Tenesa, A.; Meyer, B.F.; et al. A new GWAS and meta-analysis with 1000Genomes imputation identifies novel risk variants for colorectal cancer. *Sci. Rep.* **2015**, *5*, 10442.
25. Whiffin, N.; Hosking, F.J.; Farrington, S.M.; Palles, C.; Dobbins, S.E.; Zgaga, L.; Lloyd, A.; Kinnersley, B.; Gorman, M.; Tenesa, A.; et al. Identification of susceptibility loci for colorectal cancer in a genome-wide meta-analysis. *Hum. Mol. Genet.* **2014**, *23*, 4729–4737.
26. Houlston, R.S.; Cheadle, J.; Dobbins, S.E.; Tenesa, A.; Jones, A.M.; Howarth, K.; Spain, S.L.; Broderick, P.; Domingo, E.; Farrington, S.; et al. Meta-analysis of three genome-wide association studies identifies susceptibility loci for colorectal cancer at 1q41, 3q26.2, 12q13.13 and 20q13.33. *Nat. Genet.* **2010**, *42*, 973–977.
27. Orlando G.; Law PJ.; Palin K.; Tuupanen S.; Gylfe A.; Hanninen UA.; Cajuso, T.; Tanskanen, T.; Kondelin, J.; Kaasinen, E.; et al. Variation at 2q35 (PNKD and TMBIM1) influences colorectal cancer risk and identifies a pleiotropic effect with inflammatory bowel disease. *Hum. Mol. Genet.* **2016**, *25*, 2349–2359.
28. Jia, W.H.; Zhang, B.; Matsuo, K.; Shin, A.; Xiang, Y.B.; Jee, S.H.; Kim, D.H.; Ren, Z.; Cai, Q.; Long, J.; et al. Genome-wide association analyses in East Asians identify new susceptibility loci for colorectal cancer. *Nat. Genet.* **2013**, *45*, 191–196.
29. Zeng, C.; Matsuda, K.; Jia, W.H.; Chang, J.; Kweon, S.S.; Xiang, Y.B.; Shin, A.; Jee, S.H.; Kim, D.H.; Zhang, B.; et al. Identification of Susceptibility Loci and Genes for Colorectal Cancer Risk. *Gastroenterology* **2016**, *150*, 1633–1645.
30. Dunlop, M.G.; Dobbins, S.E.; Farrington, S.M.; Jones, A.M.; Palles, C.; Whiffin, N.; Tenesa, A.; Spain, S.; Broderick, P.; Ooi, L.Y.; et al. Common variation near CDKN1A, POLD3 and SHROOM2 influences colorectal cancer risk. *Nat. Genet.* **2012**, *44*, 770–776.
31. Tomlinson, I.P.; Webb, E.; Carvajal-Carmona, L.; Broderick, P.; Howarth, K.; Pittman, A.M.; Spain, S.; Lubbe, S.; Walther, A.; Sullivan, K.; et al. A genome-wide association study identifies colorectal cancer susceptibility loci on chromosomes 10p14 and 8q23.3. *Nat. Genet.* **2008**, *40*, 623–630.
32. Tomlinson I.; Webb E.; Carvajal-Carmona L.; Broderick P.; Kemp Z.; Spain S.; et al. A genome-wide association scan of tag SNPs identifies a susceptibility variant for colorectal cancer at 8q24.21. *Nat. Genet.* **2007**, *39*, 984–988.
33. Zhang B.; Jia WH.; Matsuda K.; Kweon SS.; Matsuo K.; Xiang YB.; Shin, A.; Jee, S.H.; Kim, D.H.; Cai, Q.; et al. Large-scale genetic study in East Asians identifies six new loci associated with colorectal cancer risk. *Nat. Genet.* **2014**, *46*, 533–542.

34. Wang, H.; Burnett, T.; Kono, S.; Haiman, C.A.; Iwasaki, M.; Wilkens, L.R.; Loo, L.W.; Van Den Berg, D.; Kolonel, L.N.; Henderson, B.E.; et al. Trans-ethnic genome-wide association study of colorectal cancer identifies a new susceptibility locus in VTI1A. *Nat. Commun.* **2014**, *5*, 4613.
35. Tenesa, A.; Farrington, S.M.; Prendergast, J.G.; Porteous, M.E.; Walker, M.; Haq, N.; Barnetson, R.A.; Theodoratou, E.; Cetnarskyj, R.; Cartwright, N.; et al. Genome-wide association scan identifies a colorectal cancer susceptibility locus on 11q23 and replicates risk loci at 8q24 and 18q21. *Nat. Genet.* **2008**, *40*, 631–637.
36. Schumacher, F.R.; Schmit, S.L.; Jiao, S.; Edlund, C.K.; Wang, H.; Zhang, B.; Hsu, L.; Huang, S.C.; Fischer, C.P.; Harju, J.F.; et al. Genome-wide association study of colorectal cancer identifies six new susceptibility loci. *Nat. Commun.* **2015**, *6*, 7138.
37. Study, C.; Houlston, R.S.; Webb, E.; Broderick, P.; Pittman, A.M.; Di Bernardo, M.C.; Lubbe, S.; Chandler, I.; Vijayakrishnan, J.; Sullivan, K.; et al. Meta-analysis of genome-wide association data identifies four new susceptibility loci for colorectal cancer. *Nat. Genet.* **2008**, *40*, 1426–1435.
38. Broderick, P.; Carvajal-Carmona, L.; Pittman, A.M.; Webb, E.; Howarth, K.; Rowan, A.; Lubbe, S.; Spain, S.; Sullivan, K.; Fielding, S.; et al. A genome-wide association study shows that common alleles of SMAD7 influence colorectal cancer risk. *Nat. Genet.* **2007**, *39*, 1315–1317.
39. Bodo, S.; Colas, C.; Buhard, O.; Collura, A.; Tinat, J.; Lavoine, N.; Guilloux, A.; Chalastanis, A.; Lafitte, P.; Coulet, F.; et al. Diagnosis of Constitutional Mismatch Repair-Deficiency Syndrome Based on Microsatellite Instability and Lymphocyte Tolerance to Methylating Agents. *Gastroenterology* **2015**, *149*, 1017–1029.

© 2019 by the authors. Licensee MDPI, Basel, Switzerland. This article is an open access article distributed under the terms and conditions of the Creative Commons Attribution (CC BY) license (http://creativecommons.org/licenses/by/4.0/).
